# Functional Changes in Pulmonary Arterial Endothelial Cells Associated with BMPR2 Mutations

**DOI:** 10.1371/journal.pone.0106703

**Published:** 2014-09-04

**Authors:** Hu Wang, Ruirui Ji, Jie Meng, Qiqiong Cui, Wenxin Zou, Lei Li, Guoliang Wang, Li Sun, Zhaohui Li, Lei Huo, Yuxin Fan, Daniel J. Penny

**Affiliations:** 1 Section of Cardiology, Department of Pediatrics, Texas Children's Hospital, Baylor College of Medicine, Houston, Texas, United States of America; 2 Cardiovascular Clinical Research Core, Texas Children's Hospital, Baylor College of Medicine, Houston, Texas, United States of America; 3 Department of Pathology, The University of Texas, MD Anderson Cancer Center, Houston, Texas, United States of America; Chinese Academy of Medical Sciences, China

## Abstract

Pulmonary arterial hypertension (PAH) is a devastating disease characterized by abnormal remodeling of small, peripheral pulmonary arteries. Germline mutations in the bone morphogenetic protein receptor type 2 (BMPR2) gene are a major risk factor for developing PAH. At present, the correlation between the BMPR2 mutation and the patient's prognosis remains controversial despite several investigations. In this study, we explored the functional effects of four BMPR2 mutations to dissect the functional significance of the BMPR2 gene defect. Cellular immunofluorescence assay of four mutants (Tyr67Cys, Thr268fs, Ser863Asn, and Gln433X) revealed that the BMPR2 protein containing Thr268fs, Ser863Asn, or Gln433X exhibited abnormal subcellular localization. The BrdU incorporation and TUNEL assay suggested that any of the BMPR2 mutations Thr268fs, Ser863Asn, or Gln433X could improve endothelial cell apoptosis and decrease cell proliferation. All of the four mutants could inhibit nitric oxide (NO) synthesis in HLMVE cells, and ET-1 levels increased in the cells transfected with mutant Ser863Asn. Our results will improve the understanding of the genotype-phenotype correlations and mechanisms associated with BMPR2 mutations.

## Introduction

Pulmonary arterial hypertension (PAH) is a rare but devastating disease characterized by pulmonary vascular proliferation and remodeling, resulting in loss of patency of the pulmonary arteries [1]. A key event in the pathophysiology of PAH is dysregulation of endothelial-dependent regulators, including nitric oxide (NO) and endothelin (ET), which, when combined with abnormal proliferation of endothelial and smooth muscle cells and vascular remodeling, results in increased pulmonary arterial pressure and vascular resistance [1], [2]. PAH may occur in a variety of clinical contexts, including as a sporadic disease known as idiopathic PAH (IPAH) and as a familial disease that typically occurs among family members who share a common genetic predisposition.

A significant advance in understanding the pathogenesis of PAH has been the demonstration of segregation as an autosomal dominant disorder with reduced penetrance, which has been mapped to a locus on 2q31-32 [3], [4]. Germline mutations in the bone morphogenetic protein receptor type 2 gene (BMPR2), a member of the transforming growth factor (TGF)-β superfamily of transmembrane serine/threonine kinase receptors, were identified in at least 50% of familial cases and as many as 40% of sporadic cases [5]–[8]. BMPR2 is a 13-exon gene that encodes four conserved domains: extracellular domain (ECD), transmembrane domain (TM), kinase domain (KD), and cytoplasmic domain (CD). Bone morphogenic proteins (BMPs) may modulate a number of pathophysiological processes, not only in the vascular smooth muscle (VSM) but also in the endothelium, which may contribute to the development of PAH. Within the endothelium, BMP has been shown to modulate the formation of the key transmitters, NO [9] and ET [10], and to regulate endothelial cell migration [11], as well as survival and proliferation [12].

Molecular studies of BMPR2 mutations in PAH demonstrate that approximately 30% are missense mutations that occur in highly conserved amino acids and are likely to perturb ligand-receptor binding or disrupt the constitutively active functional domains of the receptor. However, most BMPR2 coding mutations are frameshift and nonsense mutations [8] or involve deletions [13]. Many of the mutations predicted to cause premature truncation are thought to trigger nonsense-mediated decay of the mutant mRNA and lead to a state of haplo-insufficiency, which may represent the predominant molecular mechanism underlying inherited predisposition to PAH [14], [15].

Previous studies have shown that patients who carry a BMPR2 mutation have worse prognoses than do non-carriers in patients with PAH [16]–[18]. Whether the type of BMPR2 mutation alters the PAH prognosis remains controversial, as studies comparing prognoses in patients with truncating and missense mutations have yielded divergent results [19], [20]. A key question concerning the clinical course is whether the different types of mutations have different effects on the function of the endothelial cell in terms of release of endothelial-dependent mediators, as well as endothelial cell migration, proliferation and survival.

Accordingly, we investigated the functional significance of different BMPR2 mutations in human lung microvascular endothelial (HLMVE) cells. We compared the effects of two missense mutations (Tyr67Cys in ECD and Ser863Asn in CD) and two truncating mutations (Thr268fs and Gln433X in KD) on the release of the key endothelial mediators, NO and ET, and on endothelial cell proliferation, migration and apoptosis.

## Materials and Methods

### Cell culture

HLMVE cells were purchased from Lonza (Walkersville, MD) and maintained in the medium and growth supplements supplied by the manufacturer (EGM-2). The supplements contained 5% fetal bovine serum (FBS), hydrocortisone, human endothelial growth factor, vascular endothelial cell growth factor, human fibroblast growth factor basic, long (R3)-insulin-like growth factor-1, ascorbic acid, and antibiotics. The medium was changed every 48 hours until 80% confluence was achieved. Cells from passages 3 to 5 were used for all experiments.

The supplements contained 5% fetal bovine serum (FBS), hydrocortisone, human endothelial growth factor (GF), vascular endothelial cell GF, human fibroblast GF basic, long (R3)-insulin-like GF-1, ascorbic acid, and antibiotics. The medium was changed every 48 hours until 80% confluence was achieved. Cells from passages 3 to 5 were used for all experiments.

### Plasmid construction

The entire coding sequence of human BMPR2 was cloned, and a wild-type, V5-tagged BMPR2 construct was generated using pcDNA3.1 plasmid. Mutant BMPR2 constructs were generated by subjecting the wild-type construct to site-directed mutagenesis, using the Agilent QuickChange protocol (Agilent Technologies, Garden Grove, CA). The sequences of the wild-type and mutant constructs were confirmed by DNA sequencing, using an ABI 377 sequencer with the Applied Biosystems Big Dye Terminator v.3.1 Cycle Sequencing Kit (Foster City, CA).

### Immunofluorescent staining

Immunofluorescent staining was performed to examine subcellular localization of wild-type and four BMPR2 mutants. Briefly, HEK293 cells were cultured in two well chamber slides to 30% confluence. Cells then were transfected with BMPR2 wild-type or mutant plasmids using lipofectamine 2000 (Invitrogen, Carlsbad, CA). After 24 hours, the cells were washed with cold phosphate buffered saline (PBS) and fixed in PBS-buffered 4% paraformaldehyde at room temperature for 10 minutes and blocked with CAS-BLOCK (Invitrogen, Carlsbad, CA) for 30 minutes. The cells then were incubated for 2 hours with a mouse monoclonal antibody against V5. After being washed with PBS, cells were incubated with fluorescein isothiocyanate (FITC)-conjugated donkey anti-mouse IgG for 1 hour. Nuclei were counterstained with 4',6-diamidino-2-phenylindole (DAPI). Cells then were mounted with aqueous mounting media and examined using an immunofluorescence microscope.

### Brdu incorporation *in vitro*

To analyze cell proliferation, incorporation of bromodeoxyuridine (Brdu) *in vitro* was measured using a Brdu labeling and detection kit (Millipore, Hayward, CA). HLMVE cells were cultured in the medium containing growth supplements; the cells then were transfected at 80% confluence with BMPR2 wild-type or mutant plasmids using a Nucleofector HLMVEC-L transfection reagent and the Nucleofector 2b Device (Lonza, Walkersville, MD) according to the manufacturer's instructions. After being incubated for 48 hours at 37°C, the media were supplemented with 10 M Brdu and incubated for an additional 6 hours. The cells then were stained with a peroxidase-labeled antibody against BrdU. The absorbance of the samples at 450 nm was measured using a microplate reader.

### Apoptosis assay

Apoptotic HLMVE cells transfected with BMPR2 wild-type or mutant plasmids were determined by morphological changes. The transfected cells were cultured in serum-free medium for 24 hours and then stained with the terminal deoxyribonucleotidyl transferase (TdT)-mediated dUTP-digoxigenin nick end labeling (TUNEL) reagent (Roche, Indianapolis, IN) for in situ apoptosis detection. In brief, fixed cells were incubated for 60 minutes in a nucleotide mixture containing fluorescein-12-dUTP and TdT, according to the manufacturer's instructions. Positive controls were pretreated with 10 U/ml DNase, and negative controls were incubated without TdT. Six fields per slide were examined in each experiment. Nuclei were examined for apoptotic morphology and staining with DAPI and TUNEL reagent.

### Endothelin-1 measurement by ELISA

The cells were grown to 70% confluency and then transfected with wild-type plasmid and four BMPR2 mutants. After 8 hours, the transfection medium was replaced with fresh complete EGM-2MV media. The cell culture media were collected and stored at −80°C. The media subsequently were thawed, and ET-1 levels were measured using high sensitivity enzyme-linked immunosorbent assay (ELISA) Quantikine kits (R&D Systems, Minneapolis, MN) according to the manufacturer's protocol.

### Total nitric oxide production measurement

NO levels were estimated using the NO quantitation kit (R&D Systems, Minneapolis, MN). The cell culture medium was filtered through a 10,000-Da micropore filter prior to performing the assay. The kit was used according to the manufacturer's instructions, and ELISA was used to measure NO levels.

### Statistical analysis

All data were expressed as Mean ± SD. Differences among groups were examined for statistical significance using one-way ANOVA. A *P*-value less than 0.05 denoted the presence of a statistically significant difference.

## Results

### Subcellular localization of wild-type and mutant BMPR2 in HEK293 cells

**Figure 1:**
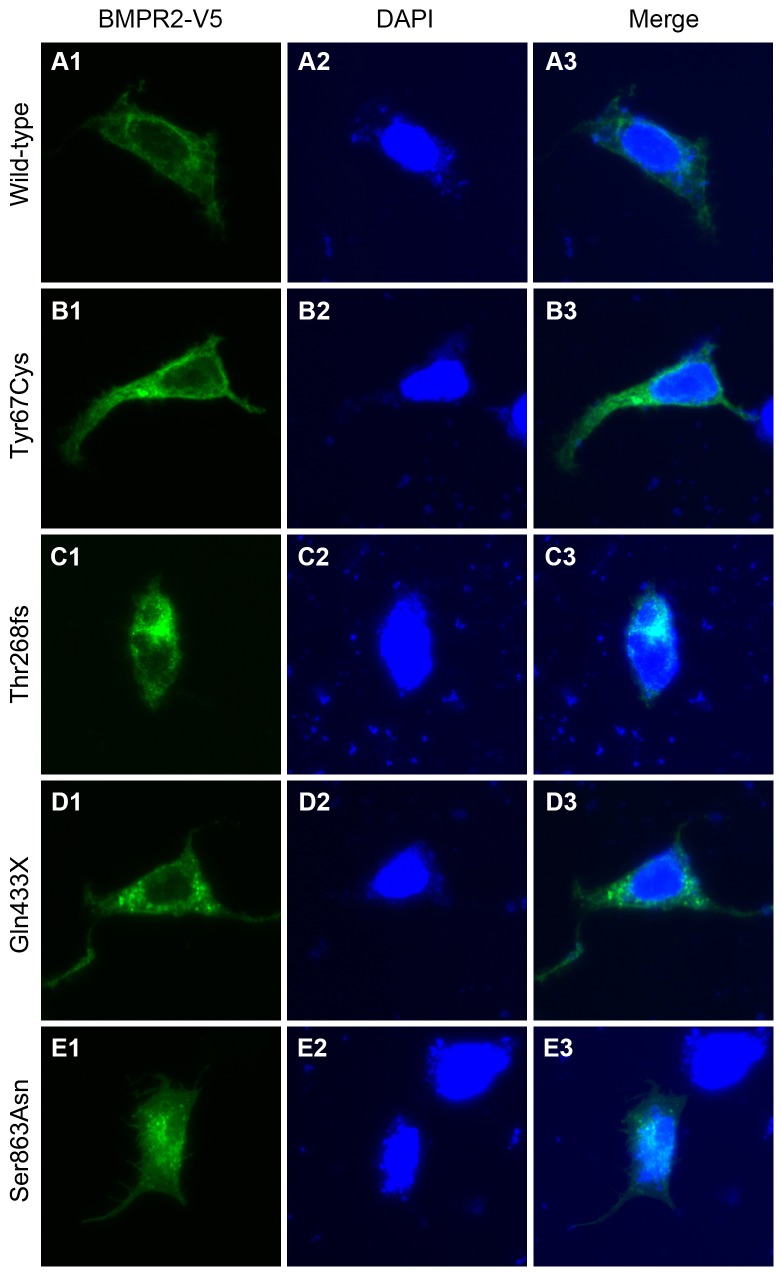
Differential subcellular localization of wild-type and mutant BMPR2. Subcellular distribution of V5-tagged wild-type (A), Tyr67Cys (B), Thr268fs (C), Gln433X (D), and Ser863Asn (E) BMPR2 mutants in transfected HEK293 cells. Permeabilized cells were subjected to immunofluorescence (fluorescein isothiocyanate; green) staining and observation by fluorescent microscopy after nuclear staining with DAPI (blue).

We examined the subcellular localization of wild-type and four mutants of BMPR2: two missense mutants (Tyr67Cys and Ser863Asn) and two truncating mutants (Thr268fs and Gln433X). The immunofluorescence staining revealed the wild-type (Figure 1A) and Tyr67Cys (Figure 1B) mutant localizing in the ECD of BMPR2, exhibited by intense staining of the cytoplasm and the plasma membrane. In contrast, the Thr268fs and Gln433X mutants, carrying a truncating mutation within the KD, were located mainly in the nucleus and abnormally aggregated in the cytoplasm, respectively (Figure 1C–D). The Ser863Asn mutant, localizing in the CD, was evenly distributed in the nucleus and cytoplasm (Figure 1E).

### Effects of BMPR2 mutations on HLMVE cell proliferation

**Figure 2:**
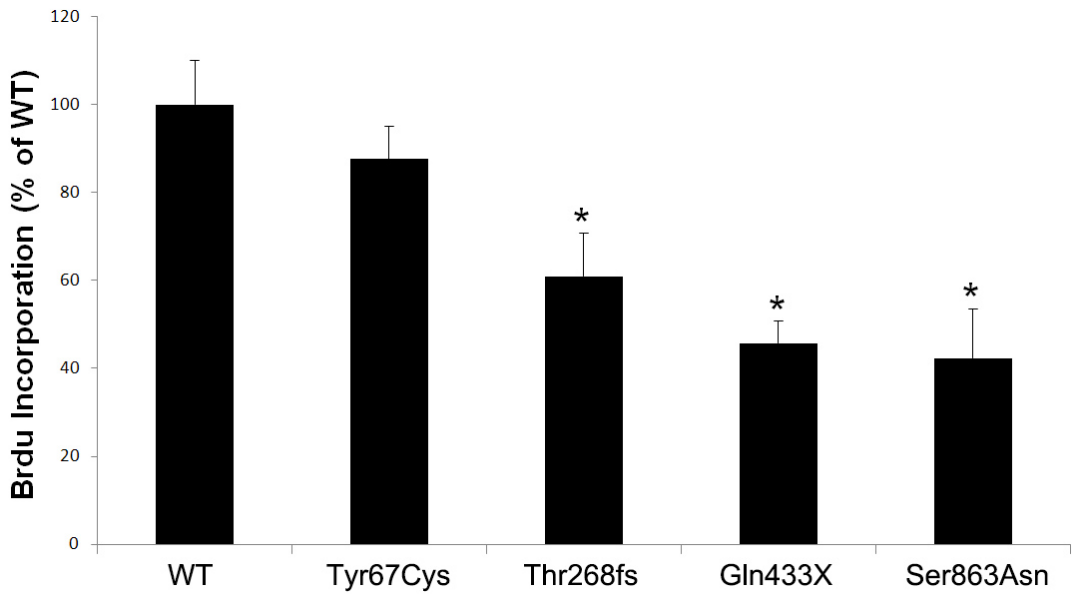
The effects of BMPR2 mutations on proliferation of human lung microvascular endothelial (HLMVE) cells. The HLMVE cells transfected with BMPR2 wild-type (WT) or mutant constructs were cultured for 48 hours in medium with fetal bovine serum and growth supplements, then labeled with BrdU for an additional 6 hours. BrdU incorporation was presented as % of WT. * *P*<0.05 compared with WT.

The HLMVE cells were transfected with BMPR2 wild-type or mutant constructs, and the cell proliferation was analyzed by incorporating Brdu. The results showed that the HLMVE cells transfected with mutants Thr268fs, Gln433X, or Ser863Asn demonstrated significantly decreased cell proliferation compared with cells transfected with wild-type BMPR2 (Figure 2). However, no significant difference in cell proliferation was observed between the cells transfected with mutant Tyr67Cys and those with wild-type BMPR2.

### Effects of BMPR2 mutations on HLMVEC apoptosis

**Figure 3:**
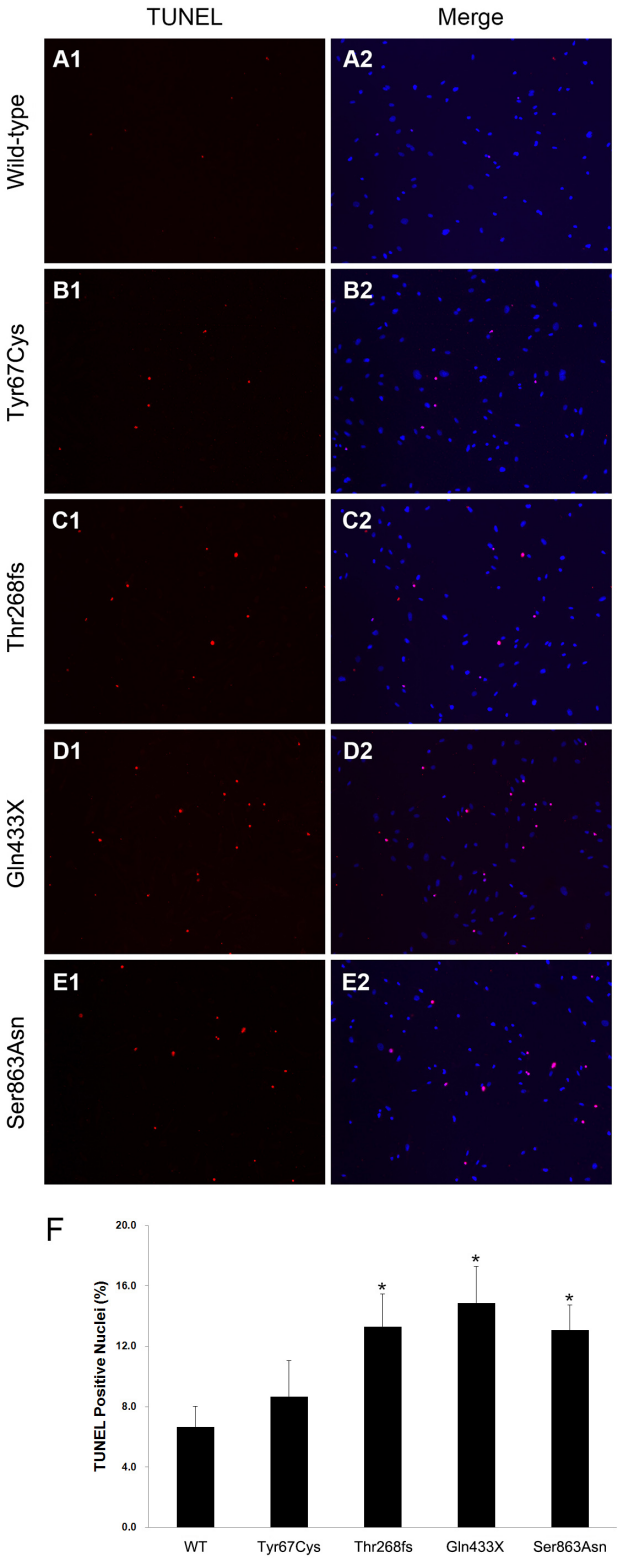
The effects of BMPR2 mutations on HLMVE cell apoptosis. Imunofluorescent images of HLMVE cells transfected with BMPR2 wild-type (WT) or mutant plasmids exposed to serum-free medium for 24 hours. TUNEL-positive cell nuclei exhibit bright red fluorescence as opposed to the blue fluorescence of the DAPI nuclear counterstain, whereas co-localization of TUNEL and DAPI staining appears as pink. B, Summary data showing the proportion of TUNEL-positive nuclei in HLMVE cells transfected with BMPR2 wild-type or mutant plasmids. *P<0.05 compared with wild-type.

To examine the effects of the four BMPR2 mutations on HLMVE cell apoptosis, we performed the TUNEL assay on the cells transfected with BMPR2 wild-type or mutant constructs. As shown in Figure 3, the number of TUNEL-positive cells was significantly increased in the cells transfected with mutants Thr268fs, Gln433X, or Ser863Asn compared with the BMPR2 wild-type transfected cells. However, no effect of mutant Tyr67Cys on cell apoptosis was observed.

### Effects of BMPR2 mutations on HLMVEC NO and ET-1 synthesis

**Figure 4:**
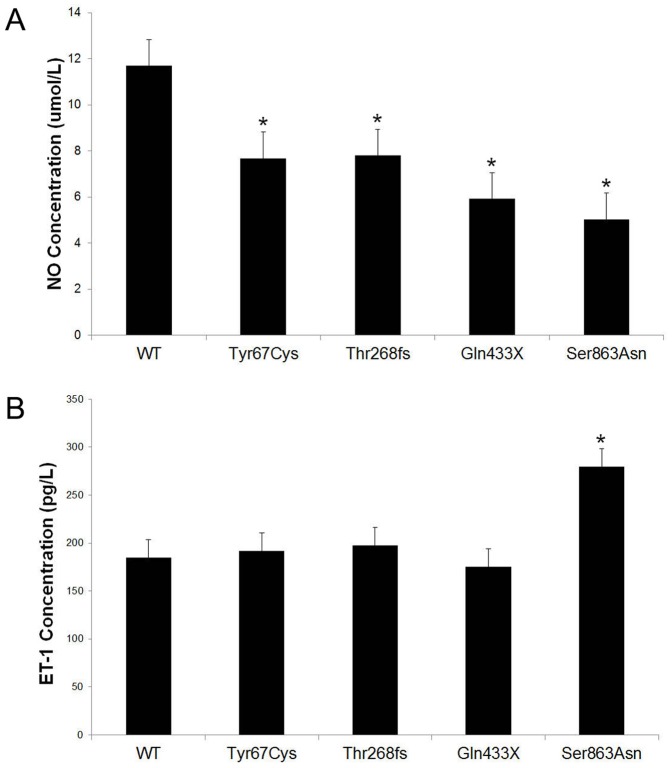
The effects of BMPR2 mutations on HLMVE cells NO and ET-1 synthesis. The HLMVE cells were transfected with BMPR2 wild-type or mutant constructs, and the levels of NO (A) and ET-1 (B) synthesis were measured by ELISA kit after 48h of transfection. *P<0.05 compared with wild-type.

The HLMVE cells were transfected with BMPR2 wild-type or mutant constructs, and the levels of NO and ET-1 synthesis were measured by ELISA kit. The results showed that the HLMVE cells transfected with mutants Tyr67Cys, Thr268fs, Gln433X, or Ser863Asn demonstrated significantly decreased NO synthesis compared with cells transfected with wild-type BMPR2 (Figure 4A). ET-1 levels significantly increased in the supernatants from the HLMVE cells transfected with Ser863Asn, but no effects of mutants Tyr67Cys, Thr268fs or Gln433X on ET-1 synthesis were observed (Figure 4B).

## Discussion

PAH is a rare and fatal disorder, with an estimated incidence of 1 to 2 per million cases per year. Pathological features of PAH appear in small pulmonary arteries and include intimal fibrosis, distal localization and proliferation of VSM cells, and pulmonary arterial occlusion [21]. The prognosis of PAH remains poor despite recent advances in therapeutic approaches that appear to prolong survival in some patients with PAH [22]. The pathogenesis of PAH is largely unknown, but ample evidence implicates the involvement of diverse vascular effectors, such as hormones, growth factors, neurotransmitters, and environmental stressors, that induce pulmonary vascular constriction, cell proliferation, or remodeling [23].

The identification of the association between mutations of the BMPR2 receptor and idiopathic PAH, both familial and sporadic, represents a major advance toward an understanding of the complex pathogenic mechanisms that underlie this fatal disease [6], [24], [25]. The BMPR2 mature protein harbors four discrete functional domains: 1) an extracellular ligand-binding domain encoded by exons 1–3; 2) a TM domain generated by exon 4; 3) a serine-threonine KD from exons 5–11; and 4) a very large intracellular C-terminal domain of unknown function from exons 12 and 13, which presents only in BMPR2 within the TGF-β receptor superfamily [7]. In common with other TGF-β receptors, BMPR2 transduces signals by forming heterodimers at the cell surface with a corresponding type I BMP receptor (BMPR1A or BMPR1B). In the presence of ligand, the serine-threonine kinase activity initiates a signal transduction cascade that involves phosphorylation of a family of signaling proteins known as Smads, and then the signal via a restricted set of receptor-mediated Smads translocates to the nucleus and regulates target-gene transcription [26], [27].

To explore the functional consequences of BMPR2 mutations located at the different domains, we generated four mutants of BMPR2 identified in patients with PAH: Tyr67Cys in the extracellular ligand-binding domain, Thr268fs and Gln433X in the serine-threonine KD, and Ser863Asn in the C-terminal domain. The last three of these mutants (Thr268fs, Gln433X, and Ser863Asn) showed abnormal subcellular localization, suggesting that the loss of function of certain BMPR2 mutants is due at least in part to their altered subcellular localization. The results also showed that the three mutants (Thr268fs, Gln433X, and Ser863Asn) could augment endothelial cell apoptosis and decrease cell proliferation. In the *in vitro* level, the effect of Tyr67Cys is different from other three mutations, which might indicate that other factors such as modifier genes and environmental changes contribute to the phenotypes in addition to the genes themselves.

Although BMPR2 is expressed by both pulmonary artery endothelial and smooth muscle cells, most investigations have focused on the potential importance of BMPR2 mutations on SMCs. A picture has emerged indicating that BMPR signaling in pulmonary VSM cells is required to maintain low VSM tone, to prevent abnormal proliferation, and to maintain normal medial structure [28]–[30]. However, the recent studies also implicate the endothelium as a critical target in the molecular pathogenesis of this disease. Krystyna and colleagues observed that knockdown of the BMPR2 using siRNA increased the basal level of apoptosis in normal human pulmonary artery endothelial cells [31]. Moreover, attenuation of BMP signaling specifically in the endothelium by selective deletion of BMPR2 is found to be sufficient to cause pulmonary vascular remodeling and the spontaneous development of pulmonary hypertension in mice [32]. In keeping with this finding, the endothelium is the predominant site of BMPR2 expression in the normal pulmonary vessels, and normal BMP signaling is required for survival of pulmonary endothelial cells [31], [33], [34].

Microvascular endothelial dysfunction is a prominent feature of PAH [35]. The role of pulmonary microvascular endothelial cells in the pathogenesis of PAH is crucial [36]. Previous studies have shown that changes in endothelial products, including decreased levels of NO and increased levels of ET-1, occur in PAH [37], [38]. ET-1 is a potent vasoconstrictor involved in the regulation of vascular tone and implicated in hypertension [39]. NO, the primary pulmonary vasodilator, is both produced and released by the endothelium. The primary functions of NO are the regulation of vascular tone, inhibition of VSM cell proliferation, and platelet aggregation [40]. The imbalance between vasodilators and vasoconstrictors in the pulmonary circulation contributes to the narrowing of the pulmonary artery [39].

Star, et al., found that BMPR2 knockdown stimulates production of ET-1 by HLMVE cells in vitro [36]. In our study, BMPR2 missense and truncating mutations reduced NO synthesis by HLMVE cells. This result is consistent with the effect of these mutants on cell apoptosis. High pulmonary levels of ET-1 in PAH have been recognized for 20 years, and several endothelial receptor antagonist therapies are known to offer clinical benefit [41], [42]; we, therefore, measured the ET-1 levels in the supernatant of the HLMVE cells after they were transfected with BMPR2 mutants. Our results demonstrated that only the Ser863Asp mutant stimulates production of ET-1 by HLMVE cells. In contrast, no effects of mutants Tyr67Cys, Thr268fs, and Gln433X on levels of ET -1 in HLMVE cells were found. As many previous studies have demonstrated that TGF-β stimulates production of ET-1 in endothelial cells [10], the question is raised as to whether the Ser863Asp mutant increases ET-1 levels itself or stimulates TGF-β signaling, which would indirectly increase ET-1 levels. The increased levels of ET-1 associated with the Ser863Asp mutant and the decreased levels of NO in all four mutants we identified may represent a mechanism that explains how BMPR2 mutations are associated with the development of PAH. These observations may have important implications regarding the link between mutations in the BMPR2 gene and the development of disease. They also suggest that patients with mutations may be more susceptible to endothelial cell loss, which has now been implicated in various experimental models as an initiating event in the pathogenesis of PAH. However, due to technical and resource restrictions, this study has the limitation of being unable to carry out experiments in biopsy samples from PAH patients. Animal model studies also might cast more light on the effects and further verify what we found in cells.

In summary, this is the first study to compare the functional significance of missense and truncating mutations of BMPR2 in HLMVE cells. No systemic difference was observed in terms of effects on subcellular localization, ET/NO measurements, and proliferation/apopotosis after HLMVE cells were transfected with different types of BMPR2 mutations.
